# Early-onset pyridoxine-dependent epilepsy due to ALDH7A1 deficiency: the first genetically confirmed case from Palestine

**DOI:** 10.1097/MS9.0000000000004880

**Published:** 2026-04-01

**Authors:** Bushra Kh. Pujee, Ramzi Mujahed, Rahaf W. Thabaineh, Sabreen Ghayyada, Darqaa Tbaisha, Mohammed Y. Atwan, Amal M. Shawabka

**Affiliations:** a Faculty of Medicine, Palestine Polytechnic University, Hebron, Palestine; b Alia Hospital, Faculty of Medicine, Palestine Polytechnic University, Hebron, Palestine

**Keywords:** ALDH7A1, genetic epilepsy, pediatric neurology, pyridoxine-dependent epilepsy, seizures

## Abstract

**Background:**

Pyridoxine-dependent epilepsy (PDE) caused by aldehyde dehydrogenase 7 family member A1 (ALDH7A1) deficiency is a rare autosomal recessive epileptic encephalopathy. It typically presents in the neonatal period with intractable seizures responsive to high-dose vitamin B6. Atypical later-onset presentations expand the clinical spectrum. Early recognition is crucial to prevent irreversible neurodevelopmental impairment.

**Findings:**

We report a 2-year-old Palestinian girl, born to consanguineous parents, who developed neonatal metabolic acidosis and encephalopathy, recurrent seizures from early infancy, and global developmental delay. EEG revealed epileptiform discharges, and brain MRI demonstrated partial agenesis of the corpus callosum and mild dilation of the lateral ventricle. Conventional antiepileptic therapy provided no improvement.

**Results:**

Whole-exome sequencing at 1 year and 8 months identified a homozygous 9.9 kb deletion in ALDH7A1, confirming PDE. High-dose pyridoxine (150 mg/day) achieved seizure control, allowing tapering of other antiepileptics. Supportive management included electrolyte monitoring and treatment of dehydration.

**Conclusion:**

This case highlights the clinical heterogeneity of PDE-ALDH7A1, underscores the importance of early genetic testing, and demonstrates the efficacy of timely pyridoxine therapy.

## Introduction

Pyridoxine-dependent epilepsy due to aldehyde dehydrogenase 7 family member A1 deficiency (PDE-ALDH7A1) is a rare autosomal recessive epileptic encephalopathy caused by a deficiency of alpha-aminoadipic semialdehyde dehydrogenase (antiquitin)^[^1^]^. The disease is characterized by seizures that respond to high-dose vitamin B6. Most affected individuals present during the neonatal or early infancy period, although atypical later-onset presentations during early childhood have also been reported, expanding the recognized clinical spectrum^[^2^]^. The estimated incidence ranges from 1 in 400 000 to 1 in 750 000 live births^[^3^]^.

Seizures are a leading cause of pediatric emergency department visits, with the highest incidence occurring in the neonatal period, affecting approximately 4%–10% of children^[^4^]^. Early recognition and treatment are critical to prevent developmental delay and long-term neurological complications.

HIGHLIGHTS

- Pyridoxine-dependent epilepsy caused by deficiency of α-aminoadipic semialdehyde dehydrogenase (antiquitin) (PDE-ALDH7A1) is a rare autosomal recessive epileptic encephalopathy.
- The disorder leads to accumulation of neurotoxic metabolites that inactivate pyridoxal-5’-phosphate, resulting in vitamin B6-responsive seizures.
- While PDE-ALDH7A1 typically presents in the neonatal period, atypical late-onset forms can occur, expanding the clinical spectrum.
- Early genetic diagnosis and lifelong pyridoxine therapy are essential to prevent irreversible neurodevelopmental impairment.

Here, we report a child with a homozygous 9.9 kb deletion in ALDH7A1 who presented with global developmental delay and epilepsy. The early onset of seizures, accompanied by microcephaly and abnormal EEG findings, illustrates the classic presentation of PDE-ALDH7A1 and highlights the clinical heterogeneity of this condition.

This is the first reported case of PDE in Palestine, emphasizing the importance of early recognition and genetic confirmation. This work has been reported in line with the Surgical Case Report (SCARE) 2025 guidelines^[^5^]^.

## Case presentation

We present the case of a 2-year-old Palestinian girl, born to consanguineous parents. She was delivered at 36 weeks of gestation by spontaneous vaginal delivery, with a birth weight of 2700 g.

### Neonatal period

Thirty minutes after birth, she developed respiratory distress and severe metabolic acidosis, necessitating admission to the neonatal intensive care unit (NICU), where she remained for 27 days. Arterial blood gas analysis revealed severe metabolic acidosis with markedly reduced bicarbonate levels and an increased anion gap, accompanied by elevated serum lactate. Given the strong family history of mitochondrial disorders, an inborn error of metabolism was suspected. Initial metabolic workup, including serum ammonia, plasma amino acids, and urine organic acids, was unremarkable. She was treated with intravenous sodium bicarbonate infusion until normalization of arterial blood gases and was started empirically on L-carnitine, vitamin B complex, and biotin. Genetic testing was sent, with results pending at that time. Initially, she was active with an intact Moro reflex; however, by 48 hours of life, she developed encephalopathy manifested by diminished Moro reflex and poor sucking. Neurology consultation was obtained, and brain MRI was requested but could not be performed due to family refusal.

### Infancy

At 2 months of age, she was re-admitted with severe metabolic acidosis and status epilepticus secondary to hypocalcemia and vitamin D deficiency, unrelated to pyridoxine dependency. She was started on oral vitamin D supplementation (2000 IU daily) and initiated on levetiracetam for seizure control at a dose of 0.5 mL (100 mg/mL) every 12 hours for 5 days, followed by 1 mL every 12 hours for 1 month. Levetiracetam was then gradually tapered over 1 month until discontinuation.

**Figure 1.**
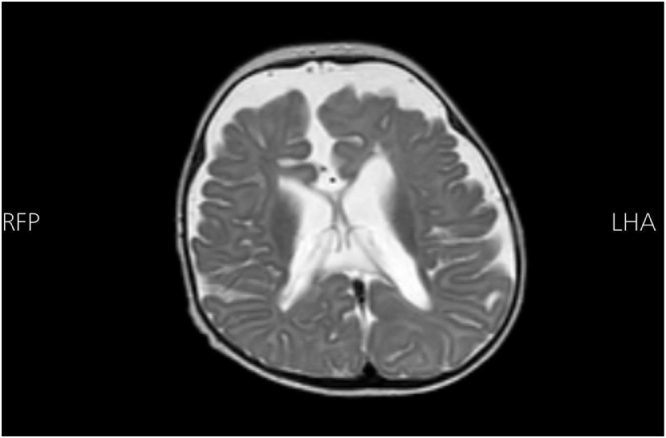
Brain MRI without contrast demonstrates partial agenesis of the corpus callosum, characterized by absence of the posterior body and splenium, while the anterior portion appears preserved. There is mild dilation of the lateral ventricles, predominantly involving the occipital horns (colpocephaly), consistent with secondary changes related to the callosal anomaly. The remaining cerebral structures, including the cortex, basal ganglia, thalami, and posterior fossa, show normal signal intensity and morphology, with no evidence of mass effect, hemorrhage, or abnormal enhancement.

At 4 months of age, a brain MRI without contrast was done and demonstrated partial agenesis of the corpus callosum and mild dilation of the lateral ventricles (Fig. 1).

At 6 months of age, she developed a generalized tonic–clonic seizure shortly after receiving the routine hexavalent (HEXA) vaccination. Acute seizure control was achieved with benzodiazepines, and maintenance therapy with levetiracetam was initiated. Laboratory evaluation did not reveal significant metabolic abnormalities. The event was considered temporally associated with vaccination, which likely acted as triggering factor rather than the underlying cause

### Development assessment

At 8 months of age, developmentally, she was unable to sit independently, roll over, or babble; however, she demonstrated a social smile and maintained visual fixation. Physical examination revealed microcephaly, encephalopathic facies, and exaggerated deep tendon reflexes.

At 10 months of age, she experienced a recurrent seizure, prompting repeat electroencephalography (EEG), which revealed diffuse background slowing with right-predominant epileptiform discharges, consistent with epileptic encephalopathy. She was treated with carbamazepine (5 mg BID), clobazam (2.5 mg BID), topiramate (25 mg once daily), and vitamin B6 (100 mg once daily). Biochemical biomarkers, including urine and plasma α-aminoadipic semialdehyde (AASA), were obtained at the time of clinical suspicion and found to be markedly elevated above the normal range. Vitamin B6 (100 mg once daily) was therefore initiated empirically due to persistent seizures unresponsive to conventional therapy and the clinical suspicion of pyridoxine-responsive epilepsy, despite the absence of prior genetic confirmation. No significant clinical improvement was observed at that time.

### Genetic tests and outcome

Given a positive family history, whole-exome sequencing was performed late at 1 year and 8 months due to financial constraints. It identified a homozygous 9.9 kb deletion in the ALDH7A1 gene (5q23.2), classified as likely pathogenic, confirming the diagnosis of PDE. High-dose pyridoxine therapy (150 mg/day) achieved seizure control, allowing gradual tapering of other antiepileptic drugs. Supportive care included close electrolyte monitoring and management of recurrent dehydration. The patient’s clinical course and management are summarized in Table 1.

**Table 1:** The key clinical events, investigations, and interventions in a child with ALDH7A1 deficiency, illustrating the progression from neonatal metabolic disturbances to refractory seizures and subsequent initiation of high-dose pyridoxine therapy.

Age	Clinical events	Investigations	Interventions
30 minutes after birth	Respiratory distress, severe metabolic acidosis	ABG, metabolic workup	IV sodium bicarbonate, NICU admission
48 hours	Encephalopathy, poor sucking	Neurology consult, Brain MRI requested	Supportive care
2 months	Metabolic acidosis, Status epilepticus	Calcium, vitamin D levels	Vitamin D, levetiracetam
4 months	MRI was requested at 48 hours of age but couldn’t be done.	Brain MRI without contrast was done	MRI revealed partial agenesis of the corpus callosum and mild dilation of the lateral ventricles
6 months	Generalized tonic-clonic seizure post-HEXA	EEG	Benzodiazepines and levetiracetam
10 months	Refractory seizure	EEG, urine, and plasma α-aminoadipic semialdehyde (AASA) biomarker	carbamazepine (5 m BID), clobazam (2.5 mg BID), topiramate (25 mg daily), and vitamin B6 (100 mg once daily) without improvement.
1 year 8 months	Suspected genetic epilepsy	Whole-exome sequencing	ALDH7A1 deficiency and high-dose pyridoxine therapy (150 mg/day) was started
Follow-up	Seizure control	Electrolytes monitoring	Gradual tapering of antiepileptic drugs, dehydration management

AASA, α-aminoadipic semialdehyde; ABG, arterial blood gas; ALDH7A1, aldehyde dehydrogenase 7 family member A1; EEG, electroencephalogram; HEXA, hexavalent vaccination; IV, intravenous; MRI, magnetic resonance imaging; NICU, neonatal intensive care unit; PDE, pyridoxine-dependent epilepsy.

## Discussion

PDE is a rare autosomal recessive condition that usually manifests as uncontrollable seizures in the neonatal period that do not improve with standard anti-epileptic medications^[^6^]^. This condition results from a deficiency of ALDH7A1^[^7^]^.

The pathogenesis of PDE-ALDH7A1 is characterized by the accumulation of toxic intermediates (AASA, Δ1-P6C, pipecolic acid) that disrupt the function of pyridoxal-5’-phosphate (PLP), which is essential for many neurological processes. Emerging evidence indicates that mitochondrial dysfunction or impaired energy metabolism also plays a role; research involving human patients and animal models (e.g., zebrafish) demonstrates abnormalities in TCA-cycle metabolites and electron transport chain activities, among others. These could underlie more severe developmental impairments^[^8^]^.

PDE-ALDH7A1 presents with a wide phenotypic spectrum, ranging from classic to atypical forms. In classic PDE-ALDH7A1, seizures typically begin in early infancy if left untreated, as observed in our patient. These seizures may present as prolonged or recurrent episodes of status epilepticus and can include a variety of types, such as focal, generalized, atonic, myoclonic seizures, and infantile spasms. In some cases, abnormal electrical activity may be seen on EEG even in the absence of overt clinical seizures. Atypical PDE-ALDH7A1, in contrast, may present later in infancy or early childhood, with seizures that initially respond to conventional anti-seizure medications before becoming refractory, and delayed pyridoxine responsiveness may occur^[^4^]^.

Microcephaly is less commonly emphasized in many PDE reports. Most literature focuses on the onset of seizure, developmental delay or intellectual disability, and imaging abnormalities. Nevertheless, severe early or poorly controlled PDE is often associated with impaired neurodevelopment and brain growth^[^1^]^.

Historically, PDE-ALDH7A1 diagnosis relied on clinical response to pyridoxine; however, immediate clinical or EEG response is not always observed and is not specific to PDE-ALDH7A1^[^9,10^]^. All affected patients show elevated plasma or urine α-AASA/Δ1-P6C, highlighting the sensitivity of these biomarkers. Mild elevations can also occur in molybdenum cofactor deficiency and isolated sulfite oxidase deficiency^[^11,12^]^. Genetic testing becomes crucial to avoid misdiagnosis and to initiate appropriate pyridoxine therapy promptly. Delayed diagnosis, as highlighted in our patient, increases the risk of irreversible neurodevelopmental impairment.

Management of PDE-ALDH7A1 is primarily based on lifelong pharmacologic pyridoxine supplementation, as affected individuals are metabolically dependent on vitamin B6. Dosages are age-adjusted, with newborns receiving 100 mg/day, infants 30 mg/kg/day (maximum 300 mg/day), and children or adults 30 mg/kg/day (maximum 500 mg/day)^[^1^]^. During acute illnesses, the pyridoxine dose may be temporarily doubled to prevent seizure exacerbation. Once seizures are controlled with pyridoxine, anti-seizure medications can often be withdrawn. Long-term therapy must balance efficacy with safety, as doses above 500 mg/day risk reversible sensory neuropathy^[^13^]^. Adjunctive strategies include lysine-restricted diets and L-arginine supplementation, which together with pyridoxine (triple therapy) have been shown to improve neurodevelopmental outcomes^[^14,15^]^. When these therapies are initiated during the first six months of life, there has been demonstrated to be a clinically significant improvement in developmental test scores^[^16^]^. Supportive care includes early intervention, individualized education plans, and access to occupational, physical, and speech therapies to address developmental delay and optimize long-term outcomes. Long-term outcomes in PDE are variable; while seizures are often controlled with pyridoxine, many patients experience developmental delays, and prognosis is poorer when treatment is initiated late or structural brain abnormalities are present^[^17^]^.

This case highlights important lessons regarding PDE-ALDH7A1. The condition has a wide clinical spectrum, and early-onset seizures should prompt consideration of this diagnosis. Early use of genetic testing and metabolic evaluation is essential, particularly in regions where cases are rarely reported. Timely recognition and initiation of pyridoxine therapy can help prevent or minimize long-term neurodevelopmental complications.

To our knowledge, this is the first genetically confirmed case of PDE-ALDH7A1 reported from Palestine. The delay in reaching a diagnosis emphasizes the importance of maintaining a high index of suspicion, particularly in children presenting with refractory seizures and unexplained developmental delay. Early recognition and timely initiation of pyridoxine therapy remain essential to optimize neurodevelopmental outcomes and to limit long-term complications.

## Data Availability

The data used to support the findings of this study are included in the article.
